# The Incidence and Demographic Distribution of Type 1 Diabetes Mellitus in Children Aged 16 or Younger Between 2000 and 2016 in Cyprus

**DOI:** 10.4274/jcrpe.galenos.2019.2019.0109

**Published:** 2020-06-03

**Authors:** Umut Mousa, Hasan Sav, Osman Köseoğluları, Ayse Şahin, Neşe Akcan, Serap Soytaç İnançlı, Rüveyde Bundak

**Affiliations:** 1 Dr. Burhan Nalbantoğlu Hospital, Clinic of Endocrinology and Metabolism, Nicosia, Cyprus; 2 Marmara Clinic, Nicosia, Cyprus; 3 Near East University, Department of Pediatric Endocrinology, Nicosia, Cyprus; 4 Private Practice, Nicosia, Cyprus

**Keywords:** Type 1 diabetes, incidence rate, Cyprus

## Abstract

**Objective:**

Type 1 diabetes (T1D) is a disease characterized by severe insulin deficiency. In 2008 our group studied the prevalence of diabetes in adults between 20-80 years of age in Cyprus but data regarding this incidence in the pediatric population is lacking. The objective of this study was to report the incidence of T1D among permanent inhabitants aged 16 years or younger between 2001-2016 in Cyprus.

**Methods:**

This study was a retrospective analysis. The patients were mainly evaluated and recorded at Dr. Burhan Nalbantoğlu Hospital, Nicosia. Data was also reviewed from Famagusta Government Hospital, Kyrenia Government Hospital, Near East University Hospital and the Cyprus Turkish Diabetes Association.

**Results:**

A total of 107 subjects were diagnosed as T1D between 2001 and 2016 in the pediatric age group. Forty-nine (45.7%) were girls and 58 (54.3%) were boys. Of these 38.7% were resident in Nicosia, 30.2% Famagusta, 12.3% Kyrenia, 9.4% Guzelyurt and 7.5% Iskele. The proportion of newly diagnosed T1D was highest among children aged 9-12 years (35.5%) followed by children aged 5-8 years (32.7%). Newly diagnosed T1D most frequently presented in March and April. The overall mean incidence rate was 11.1/100,000 between 2001 and 2016. The incidence rates were similar and comparable among the years.

**Conclusion:**

This study is the first to analyze the incidence of T1D in Cyprus. Compared to other countries the incidence rate is intermediate. Our findings are similar to the incidence rates of T1D in South Cyprus and Turkey.

**What is already known on this topic?**

The incidence of type 1 diabetes (T1D) has been reported in various countries. There is no data regarding the incidence rate of T1D in Cyprus.

**What this study adds?**

This study is the first to report the incidence of T1D in the pediatric population from Cyprus. The overall incidence was 11.1/100,000 between 2001 and 2016, which is an intermediate incidence compared to other countries, and did not differ from year to year.

## Introduction

Type 1 diabetes (T1D) arises from the autoimmune destruction of pancreatic β-cells leading to a life-long dependence on exogenous insulin (1). The disease most commonly presents in children and adolescents (2). T1D is also the most frequently encountered chronic disease of childhood (3).

The incidence and prevalence of this disorder are not uniform worldwide. A large variability has been reported among different populations. Seasonal variations have also been reported. The highest incidence is observed in Scandinavian countries, whereas China and countries close to the equator have lower incidences (4,5,6).

Cyprus is an island located in the Mediterranean region. A study published in 2012 reported the incidence of T1D in South Cyprus. The authors reported that the overall mean incidence rate was 12.46/100,000 between 1990 and 2009. In the first study decade, the mean incidence rate was 10.8/100,000. However, in the second study decade, the mean incidence rate was 14.4/100,000. Thus, the authors concluded that the incidence of T1D was rising in Cyprus (6).

Sardinia is another Mediterranean island which has a higher incidence of T1D than expected from the geographical region. The incidence rate was reported as 38.8/100,000 between 1989 and 1999 (5).

According to a diabetes survey which our group performed in 2008, the prevalence of diabetes between 20-80 years was 11% and prediabetes 18% in Cyprus. However, studies regarding the pediatric population are lacking (7).

In this study, we aimed to calculate the incidence rates of T1D in Cyprus in subjects 16 years of age or younger between 2001 and 2016.

## Methods

The study was approved by the Dr. Burhan Nalbantoğlu Hospital Ethical Committee with research number: 026/19. The study was retrospective and did not involve interventions, thus we did not obtain informed consent from the patients or their parents.

Cyprus is divided into five main districts. These are Nicosia, Kyrenia, Famagusta, Iskele, and Guzelyurt. According to data obtained from the obligatory population survey covering the whole of Cyprus, which took place in 2011, a total of 59,315 permanent inhabitants are present between 0-16 years of age. The distribution of this population among districts is seen in Table 1 (8).

Dr. Burhan Nalbantoğlu Hospital, in Nicosia, is the only government hospital which has an endocrinology clinic. Thus, all pediatric patients with hyperglycemia are transferred to this department. Data was collected from this hospital together with records from Girne Government Hospital, Famagusta Government Hospital, Cengiz Topel Government Hospital, and Near East University Hospital. Subjects are advised to register with and provide records to the Cyprus Turkish Diabetes Association, which is a Non-Governmental Organization. The data of the Cyprus Turkish Diabetes Association was used to cross-reference and confirm data collected from hospital records.

T1D was diagnosed according to the International Society for Pediatric and Adolescent diabetes 2018 clinical practice consensus guidelines (9).

Subjects eligible for the study included:

1) Turkish Cypriots and permanent inhabitants in Cyprus;

2) Those with a fasting blood glucose ≥126 mg/dL, low c peptide levels and at least one positive antibody (insulin antibody, islet antibody, glutamic acid decarboxylase antibody);

3) Aged 16 years or younger;

4) Did not have or were not suspected to have type 2 diabetes, monogenic diabetes, neonatal diabetes and other types of secondary diabetes.

Data collected on each case included the gender of the subject, the age of the subject at diagnosis, the year and month of diagnosis and the district in which the subject was resident.

### Statistical Analysis

Analysis was performed using the Statistical Package for Social Sciences, version 17 (IBM Inc., Armonk, NY., USA). The incidence was calculated by using the number of cases reported each year by age group (0-4, 5-8, 9-12, 13-16) and sex (male or female). The incidence rates were calculated per 100,000 heads of the population. Comparison of proportions and incidence rates were performed via the χ^2^ test. A p value <0.05 was considered statistically significant.

## Results

According to records, a total of 107 new cases of T1D were identified between 2001 and 2016 in children and adolescents younger than 16. Of these 49 (45.7%) were girls and 58 (54.3%) were boys. The male/female ratio was 1.18:1. The median age of diagnosis was 9 years overall and was the same for both genders. According to the population survey of 2011, the total population of subjects aged 16 or younger was 59,315. Out of these 30,561 were boys and 28,754 were girls. The mean annual incidence rates for boys was 11.86/100000 and for girls 10.65/100000.

The proportion of newly diagnosed T1D was highest among children aged 9-12 years (35.5%) followed by children aged 5-8 years (32.7%). Thus the highest incidence rate was in the 9-12 year-old age group (17.6/100,000) while the lowest incidence by age was found in children younger than or equal to 4 years (5.75/100,000). Proportions and incidence rates according to age groups are seen in Table 2.

**Figure 1:**
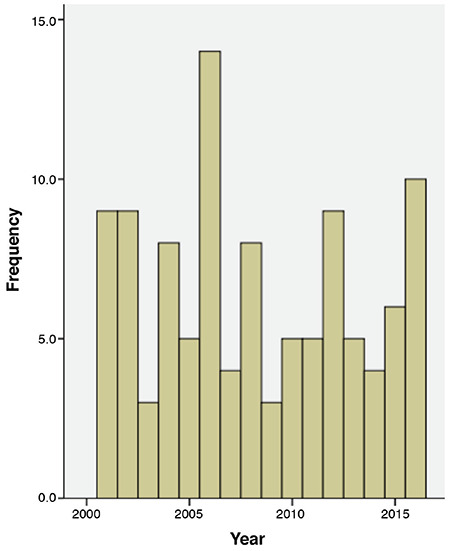
Incidence of type 1 diabetes by years (2000-2016)

The number of cases per year is seen in Figure 1. The overall mean incidence rate was calculated as 11.1/100000 between 2001 and 2016. Between 2001-2005 the mean incidence rate was 11.2/100000; between 2006-2010: 11.4/100000; between 2011-2016: 10.8/100000. The mean incidence rates were statistically similar across the three periods (p>0.05).

The highest population effect of T1D was in Nicosia. However, the highest incidence rate was observed in Famagusta (14.2/100,000) and the lowest incidence rate was observed in Kyrenia (5.8/100,000) The incidence rates of T1D in Kyrenia was significantly lower than the other districts (p<0.05). The incidence rate in Famagusta was similar to that of Nicosia (p>0.05), and higher than that of the other districts (p<0.05) (Table 1).

**Figure 2:**
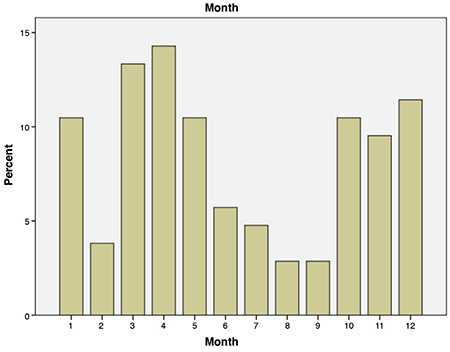
The diagnosis of type 1 diabetes according to the month of diagnosis

Newly diagnosed T1D most frequently presented in April and March (14.3%, 13.2% respectively) and higher incidences were generally seen in the winter months compared to the summer months. The monthly distribution according to time of T1D diagnosis is seen in Figure 2.

## Discussion

In this study, the incidence of T1D in Cyprus was investigated. The results show that the mean annual incidence is intermediate (11.1/100,000) in children and adolescents aged 16 or younger (10). In Cyprus, there is only one other study of pediatric T1D incidence, which was undertaken in Southern Cyprus (6). This study is the first population-based report on T1D incidence in the pediatric population of Cyprus.

The study of Skordis et al (6) (2011) analyzed a 20-year data on the incidence of T1D in subjects aged 15 or younger in the Southern Cyprus Territory. In the first decade of the study, the incidence was reported as 10.8/100,000, whereas the incidence was 14.4/100,000 in the second decade. Thus the authors concluded that the incidence of T1D was rising. Our results were not compatible with these findings. We compared the incidence rates between 2001- 2008 and 2009-2016. The mean incidence rate was 11.3/100,000 between 2001-2008 and 11.1/100,000 between 2009-2016. We also report that between 2001-2005 the mean incidence rate was 11.2/100,000; between 2006-2010: 11.4/100000 and between 2011-2016: 10.8/100,000. Thus, the distribution of incidences was balanced among the years.

Cyprus has close relations with Turkey and immigration in both directions is not rare. A prospective study which was published recently in Turkey calculated the mean crude annual incidence of pediatric T1D as 8.99/100,000 between 2013 and 2015 (2). A retrospective study analyzing the incidence of T1D in children 14 years or younger, in Southeast Turkey calculated the mean incidence as 7.2/100,000 (3). A nationwide study involving 17,175 prevalent cases of T1D in subjects younger than 18 years calculated the age-standardized incidence rate as 10.8/100,000 (95% confidence interval: 10.1-11.5) in Turkey (10). Our figures are between those of South Cyprus and Turkey.

Cyprus is an island in the Mediterranean Sea. It has been argued that there is a climate effect on the incidence rate of T1D. We compared our data with other Mediterranean countries having data on the incidence rate of T1D. In Eastern Sicily, the incidence rate of T1D was 10.1/100,000 between 1989 and 1990, and 11.7/100,000 between 1990 and 1994 (4). Our results are similar to the data reported from eastern Sicily.

In Malta, the mean annual incidence rate of T1D was 13.6/100,000 between 1980 and 1987 (11), and 24.68/100,000 between 2006 and 2010 in children aged 14 or younger. The authors calculated a mean annual increase in the incidence of T1D of 21.8% per year (12). The island of Sardinia has also reported high incidence rates; the mean incidence rate was reported as 38.8/100,000 between 1989 and 1999 (13). All these Mediterranean islands are relatively lowly populated which could decrease the reliability of calculated incidence rates.

According to our data, the mean annual incidence rate was slightly higher in boys compared to girls. Previous studies have reported a male predominance in high incidence countries and a female predominance in low incidence populations. A study by Karvonen et al (14) reported that 88% of low incidence populations were predominantly girls, and high incidence countries were predominantly boys. Data from Sardinia report a male/female ratio of 1.4 in children aged 15 or younger (13). Our results were similar to the data reported in Southern Cyprus which reported a male/female ratio of 1.06/1 (6).

The proportion of newly diagnosed T1D was highest in the 9-12 years age group followed by the 5-8 years age group. The lowest incidence rate was found in the 0-4 years age group. These findings are consistent with previous large scale studies. A Multinational study (DIAMOND Project) documented that 10-14 year-old children had the highest incidence rates (14) whereas data from Malta reported the highest incidence rate in children aged 5-9 years (5).

In our cohort, T1D was most frequently diagnosed in April, followed by March. The study of Skordis et al (6) from Southern Cyprus reported a significantly higher incidence of T1D in cold months (November, December, January, February) compared to hot months (June, July, August, September) in the first decade of the study. Incidence rates in the remaining months were similar to that of the cold months.

The highest mean annual incidence rate was in Famagusta and the lowest rate was in Kyrenia. Both districts have Mediterranean Sea coastlines and the climates of both districts are similar. However, the area of Cyprus is too small to make any conclusions about climate effects and also the number of individuals with T1D are too small to make conclusions about differences in incidence rates among districts. Also due to loss of data, we were unable to record the districts of two subjects which can lead to up to a 2% error rate in this analysis.

### Study Limitations

The retrospective format is a study limitation. All subjects are diagnosed and treated at a single center. Thus, the study is strong due to excellent case ascertainment.

## Conclusion

In conclusion the incidence of T1D in children aged 16 years or younger is 11.1/100,000 in Cyprus which is intermediate in comparison to other Mediterranean islands. The incidence rate does not appear to be increasing when data are compared between five year periods from 2000 to 2016.

## Figures and Tables

**Table 1:**
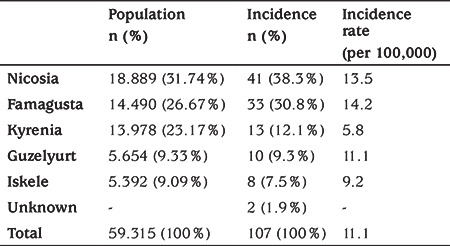
Distribution of the <16-year old population across the districts according to the 2011 population survey in Cyprus, together with proportions and incidence rates in these districts

**Table 2:**
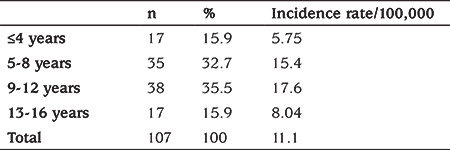
Proportions of newly diagnosed type 1 diabetes mellitus according to age groups
